# Modelling Interactions between Forest Pest Invasions and Human Decisions Regarding Firewood Transport Restrictions

**DOI:** 10.1371/journal.pone.0090511

**Published:** 2014-04-15

**Authors:** Lee-Ann Barlow, Jacob Cecile, Chris T. Bauch, Madhur Anand

**Affiliations:** 1 Department of Mathematics and Statistics, University of Guelph, Guelph, Ontario, Canada; 2 School of Environmental Sciences, University of Guelph, Guelph, Ontario, Canada; Vrije Universiteit, Netherlands

## Abstract

The invasion of nonnative, wood-boring insects such as the Asian longhorned beetle (*A. glabripennis*) and the emerald ash borer (*A. planipennis*) is a serious ecological and economic threat to Canadian deciduous and mixed-wood forests. Humans act as a major vector for the spread of these pests via firewood transport, although existing models do not explicitly capture human decision-making regarding firewood transport. In this paper we present a two-patch coupled human-environment system model that includes social influence and long-distance firewood transport and examines potential strategies for mitigating pest spread. We found that increasing concern regarding infestations (*f*) significantly reduced infestation. Additionally it resulted in multiple thresholds at which the intensity of infestation in a patch was decreased. It was also found that a decrease in the cost of firewood purchased in the area where it is supposed to be burned (*C*_l_) resulted in an increased proportion of local-firewood strategists, and a 67% decrease in *C*_l_ from $6.75 to $4.50 was sufficient to eliminate crosspatch infestation. These effects are synergistic: increasing concern through awareness and education campaigns acts together with reduced firewood costs, thereby reducing the required threshold of both awareness and economic incentives. Our results indicate that the best management strategy includes a combination of public education paired with firewood subsidization.

## Introduction

The invasion of nonnative, wood-boring insects such as the Asian longhorned beetle (*A. glabripennis*) and the emerald ash borer (*A. planipennis*) is a serious ecological and economic threat to Canadian deciduous and mixed-wood forests. These pests have the potential to cause catastrophic damage to Ontario’s deciduous and mixed forests and therefore research into their spread and control has become vital. The diversity of potential host trees–including maple, poplar, birch and willow–for the Asian longhorned beetle (ALB) has led to an estimate that if the beetle were to become established in North America, approximately 1.2 billion trees could be at risk [1]. Furthermore, between April 2005 and March 2010, the Canadian government spent $24.9 million in response to the threat of the ALB in operations, programs, science, and public affairs [2]. As its name suggests, the emerald ash borer (EAB) infests all species of ash trees found in North America. Infestation of a tree generally results in death in one to four years, depending on the health and size of the tree, as well as the degree of infestation [3]–[6]. While adult beetles may be capable of flying up to 5 km, most travel less than 100 m when ash trees are near [5] so the transport of ash firewood and other ash products serves as a critical vector for the spread of these insects to new regions [3]. The popularity of the various species of ash as shade trees means that the impact of an emerald ash borer infestation in North America would be devastating. In Ohio alone, the estimated cost of the landscape loss, removal, and replacement of all ash trees is estimated between $1.8 and $7.6 billion, or approximately $157,000–$665,000 per 1000 residents [7]. The Canadian government spent $17.6 million in response to the threat of the EAB in operations, program, science, and public affairs between April 2005 and March 2010 (personal communication, Ontario Parks).

The current literature on mathematical models of forest insect pests has focused on a specific pest or modeled a single generic pest, with various implications for pest elimination. Our deterministic model was loosely based on the ordinary differential equation model introduced in [8]. They did not examine pest control strategies, but rather found that invasion by pests into a stationary (stable) forest ecosystem can result in intensive oscillations of age structure of tree populations. [9] used differential equations to model the elimination of the ALB either before or after they have had the opportunity to reproduce. The authors found that under certain detection and elimination conditions, it was possible to eliminate the outbreak, but only when the probability of early detection and removal of infested trees was high. Two delays were built into this model to account for the delay between infestation and death of the tree as well as the maturation delay for the beetle. Additionally, the study by [10] used differential equations to model the interaction between generic tree and insect variables based on a model from [11]. The results showed that in some forests, insect outbreaks are periodic and endogenously generated while in others they are aperiodic and triggered by exogenous environmental shocks, which agrees with field studies. However, elimination strategies were not explored.

A few models have attempted to include human factors in pest-spread dynamics, including long-distance transport of pests through firewood movement. For example, [12] used statistical and gravity models to examine the probability of infestation due to natural movement of the insects and human population density, including dispersal through firewood. It was found that human population and activity were major factors in the spread of the EAB and that dispersal via diffusion was much easier to predict than jump displacement. Although human-assisted dispersal was included in this model, it was not represented mechanistically, and the model did not examine coupled feedbacks between human behavior and pest spread, wherein changes in infestation levels can influence human adherence to firewood movement restrictions.

Early tree-removal containment initiatives have been unsuccessful and current firewood quarantines are having little effect on the spread of ALB and EAB [6]. Both species tend to travel less than 2 km on their own but are easily transported large distances by humans with firewood as a vector. For this reason, the control of firewood transport has become critical in mitigating the spread of the ALB and EAB, along with other wood-boring pests. However, successful control of pest spread through firewood transport relies upon the voluntary participation of a large number of individuals, whose perceptions of the value of forest conservation and the importance of their role in it will determine whether they adhere to the restrictions or not. Education campaigns on the impact of forest pests and the role of firewood transport in their dispersal have had some success in getting individuals to adhere to firewood movement restrictions, but only when the restrictions are not too inconvenient (D.M. Runberg, unpublished report, 2011). Obviously, eliminating firewood transport would mitigate dispersal for all invasive wood-boring pests that disperse in this way, and thus have an advantage over species-specific control strategies.

Because of the inherent human behavioural aspect of this issue, coupled human-environment system models have much to offer, since human decisions both affect–and are affected by–the state of the environment. This is an aspect of the problem that previous forest pest models were not developed to address. Our study had three primary objectives: to develop a human-environment system model of the dispersal of ALB and EAB, including long-distance transport of firewood; to examine the impact of human activity on preventing dispersal, including economic factors and social learning; and to determine the best strategy to slow or stop the spread of these pests.

## Methods

### Forest Model

Many existing models depict the changes in a forest ecosystem due to the infestation of an insect pest, without including the corresponding human-environment interactions [8]–[14]. To model the effect of social factors and human-environment system interactions we will combine a simple forest population model with a model of social learning and economic payoffs. We manage the increased model complexity caused by mechanistically modelling human behaviour by opting for a simplified model of forest pest spread. The following system of equations describing a two-patch system is derived from a model used by [8], which modelled non-even-aged forests affected by insect pests
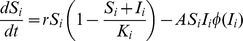





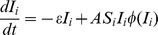
where *i = 1,2* and



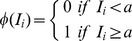

*Si* and *I_i_* represent the number of susceptible (healthy) and infested trees in patch *i* respectively, *r* is the fecundity of the trees, *K_i_* is the carrying capacity of patch *i*, *A* is the transmission rate of the pest, and ε is the fatality rate of the infested trees. The *ASiIiΦ(I_i_)* term represents the infestation of susceptible trees; we assume that susceptible trees are infested at a rate proportional to the product of the number of susceptible trees (the potential hosts) and infested trees in the patch. The function *Φ(I_i_)* represents effects that can prevent population growth when population densities are sufficiently small, such as a strong Allee effect. It also allows us to (imperfectly) capture stochastic fadeout effects that would emerge more naturally out of a fully stochastic model.

In the absence of infestation, the tree population grows to a fixed carrying capacity *K*_i_ and the growth of the healthy trees is modeled as
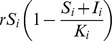



While this model is relatively simple, such simplicity is desirable in a first human-environment system model of forest pest control, since it allows us to establish basic features of such systems in a more transparent way than a model with many parameters would. However, we discuss the potential impact of such simplifications in the Discussion section. We also explored a multi-patch system, however, the results were qualitatively unchanged under a similar parameterization as for the two-patch system, so we will only discuss the two-patch system.

### Social Influence Model

Individuals are subject to multiple pressures, such as the pressure to conform to social norms, to make economically beneficial decisions, and to avoid negative consequences. We express how individuals gauge these pressures through a utility (payoff) approach, where individuals can rank their preferences for one outcome or another according to a numerical score. These pressures can be modeled using two payoff functions: one function is the payoff *P*_l_ for the “local” strategy of always buying firewood in the same patch where it is burned, and the other function is payoff *P*_t_ for the “transport” strategy of buying firewood in one patch and burning it in another patch:
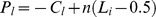





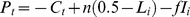
where *n* represents the strength of social norms, *C_l_* is the direct economic cost of purchasing local firewood and “burning it where you buy it”, *C_t_* is the direct economic cost of transporting firewood, which may potentially disperse pests as a result, *L*_i_ is the proportion of individuals in patch *i* who adhere to firewood movement restrictions and thus do not transport firewood to the other patches, and *f* is a proportionality constant controlling the impact of infestation levels on individual decisions. This is representative of the level of concern for causing new infestations, with higher *f* corresponding to greater concern.

In the equation for *P*_l_, when *L*_j_ >0.5, the second (social pressure) term is positive, conferring a positive payoff to those who adopt the local strategy. This corresponds to deriving a benefit from adhering to the socially dominant strategy. Similarly, for *L*_j_ <0.5, the local strategists are in the minority and hence individuals who adopt this strategy receive a penalty. A similar explanation applies to the second term in the equation for *P*_t_. Note that in the equation for *P*_t_ there is an extra penalty associated with the concern for causing a new infestation.

The rate at which an individual switches from one strategy to another is a function of the difference in payoff between the two strategies:
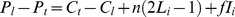



We assume that each individual samples others in the population at some rate, and switches to the sampled person’s strategy (if different from their own) with a probability proportional to the difference in payoff, if positive (i.e. if they stand to gain). Hence, the rate at which the 1-*L*_i_ transport strategists in the population sample the *L*_i_ local strategists and thereby become local strategists themselves is:
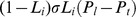
when *P*_l_ >*P*_t_, and it is zero otherwise, and where *σ* is the sampling rate times the proportionality constant, which we refer to as the imitation rate (for *σ* sufficiently small the probability of switching remains less than 100%.) Similarly, the rate at which the *L*_i_ local strategists in the population sample the 1-*L*_i_ transport strategists and become transport strategists themselves is



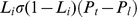
when *P*_t_ >*P*_l_, and it is zero otherwise. Adding these two equations yields the total rate of change in the proportion of local strategists:



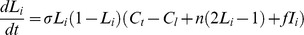



When it is more expensive to transport firewood than to buy it locally (*C*_t_-*C*_l_>0), when local strategists are in the majority (2*L*_i_−1>0), and/or when there is infestation present (*p*>0, *I*_i_>0), then the payoff for switching to the local strategy is positive and hence *L*_i_ increases over time. In contrast, when the sum of these three terms is negative, then the payoff for switching to the transport strategy is positive, hence *L*_i_ decreases over time.

### Human-environment System Model

Our goal is to determine the relationship between human decisions and the forest ecosystem and thereby suggest an effective management strategy. Hence, we must have a fully coupled system of equations. To that end, we define the impact of human decisions on the forest as serving as a vector for insect dispersal between patches through firewood transport. As our social influence model already depends on the level of infestation *I*_i_, no changes need to be made in this equation. However, we add terms to the forest growth equations capturing cross-patch infestation:
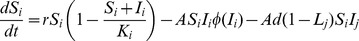





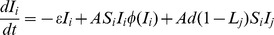





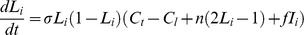
where *d*<1 is a proportionality constant controlling the rate of cross-patch infestation from patch *j* to patch *i*≠*j.*

### Parameterization

All baseline parameter values appear in Table 1. The environmental and economic parameter values used in the model were determined through a literature review [4], [7], [11], [15], [16] and through contact with Ontario Parks. The fecundity of trees is difficult to determine, particularly because we are attempting to represent a wide variety of tree species, many of which have very different fecundity rates. To resolve this problem as best as possible we used the results of a model by [16] examining the rate of seed dispersal among a tree species affected by the ALB and EAB. We used the intermediate survival rate of 0.00024 paired with the average number of trees per year β = 25000 to get a fecundity of *r* = 0.06 per year [16]. The transmission rate of the pest was determined by calculating the amount of time needed for a patch to reach 50% of full infestation and adjusting *A* to ensure that our model reflected that timeframe. The specified value of *A = *6.5×10^−4^ per year corresponds to a 5 ha patch with a infestation spread rate of 200 meters per year, which agrees with the literature [7], [15], [17]. Based on a phone survey of local parks, we assumed *C*_l_ = $6.75 and *C*_t_ = $5.00, where *C*_l_ is the typical price for a bundle in parks and is *C*_t_ is the price for a bundle often available through private vendors. Parameters *n*, *f*, and *σ* are difficult to estimate directly and hence were varied in the analysis.

**Table 1:** Parameter definitions and values.

Symbol	Definition	Value	Source
*r*	Fecundity of trees	0.06/yr	[11]
*A*	Transmission rate of pest	6.5×10^−4^/yr	[7], [11], [15]
*d*	Between-patch transmission	0.05	–
*ε*	Time to fatality for infested trees	3 yr	[4], [16]
*C_l_*	Cost of buying local firewood	$6.75	†
*C_t_*	Cost of transporting firewood	$5.00	‡
*n*	Strength of social norms	–	–
*f*	Impact of outbreak on decisions	–	–
*K_i_*	Carrying capacity in patch *i*	5000	[7]
*σ*	Imitation dynamic	–	–
*a*	Allee effect threshold	1	–

† The cost of local firewood was determined by personal communication with Ontario Parks.

‡ The cost of transporting firewood was determined by observation of costs of firewood available for private sale.

## Results

The model was analyzed through numerical simulations in the *R* programming language and the deSolve package [18]. Each of the three social influence parameters *n*, *f*, and *σ* were varied to determine their effects on the system dynamics. Each parameter was tested independently to control for the effects of interaction. Additionally, different local firewood costs *C*_l_ were tested to determine the effect of subsidizing the cost of firewood in provincial and national parks. Finally, the concern parameter *f* was adjusted while reducing local firewood costs to determine the combined effect of changing both parameters, as opposed to changing just one or the other.

### Baseline Scenario

Our baseline scenario, reflecting conditions where an infestation has just begun in one patch, and adherence to firewood movement restrictions is initially low, assumed *S_1_(0)* = 4985, *S_2_(0)* = 5000, *I_1_(0)* = 15, *I_2_(0)* = 0, *L_1_(0)* = 0.1, and *L*_2_(0) = 0.1. These initial values imply that both patches begin at carrying capacity since *S_1_*+ *I_1_* = 5000 = *K*_1_ and *S_2_*+ *I_2_* = 5000 = *K_2_*, that patch 1 has a small infestation while patch 2 is initially healthy, and that both patches start off with 10% of individuals being local strategists. This situation allows for a majority of individuals to begin as transport strategists, and hence provides a tendency for the population to continue being dominated by transport strategists unless events cause a change in the population composition. Furthermore, *n* = *f* = 0.1, and *σ* = 0.1 per year in the baseline scenario.

**Figure 1:**
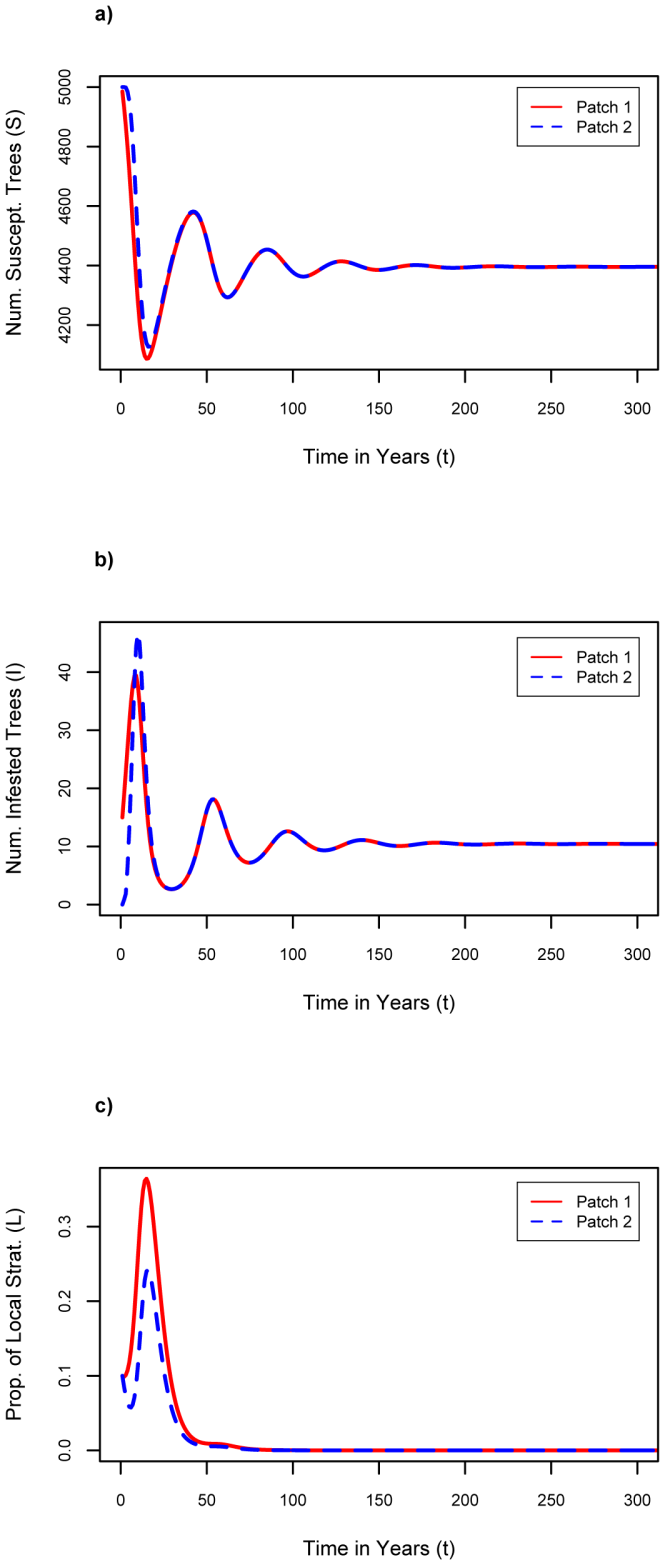
Results of baseline. Baseline: n = 0.1, f = 0.1, σ = 0.1, Cl = $6.75 where a) number of susceptible trees as a function of time; b) number of infested trees as a function of time; c) proportion of local firewood strategists as a function of time.

For the baseline parameter assumptions, we see that over time, *S_1_* and *S_2_* both equilibrate to approximately 4400 (Fig. 1a), which is below carrying capacity, and *I_1_* and *I_2_* both equilibrate to approximately 10 (see Fig. 1b), thus the initial infestation in patch 1 has spread to patch 2 and becomes endemic in both patches. The percentage of local strategists, *L_1_* and *L_2_*, initially increases in both patches due to the large initial peaks in the number of infested trees, but as the infestations become endemic, *L_1_* and *L_2_* fall back down to zero due to the effects of social pressure (Fig. 1c). We note *C*_t_<*C*_l_ at baseline values, hence economic pressures also acted against wider adoption of firewood movement restrictions.

In Figure 1, the largest oscillation corresponding to the initial infestation is over within the first decade. Oscillations that follow are drawn out for a long time period, but are relatively small in amplitude, and in real populations these oscillations would be washed out by noise and/or trends.

### Increasing the Strength of Social Norms

**Figure 2:**
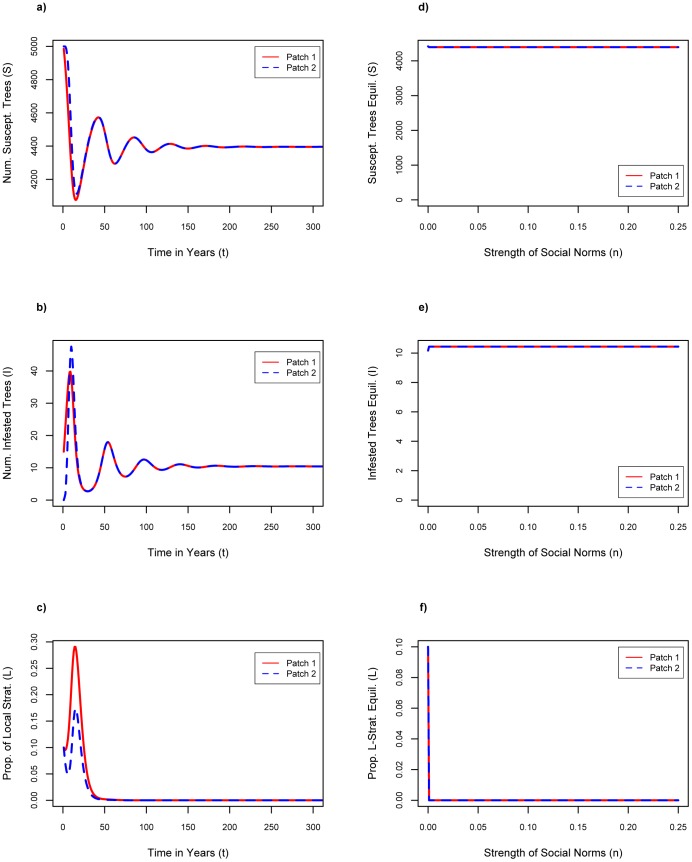
Results of increasing strength of social norms. Increase strength of social norms: f = 0.1, σ = 0.1, Cl = $6.75 where a) number of susceptible trees as a function of time with n = 0.5; b) number of infested trees as a function of time with n = 0.5; c) proportion of local firewood strategists as a function of time with n = 0.5; d) susceptible tree equilibria for increasing values of n; e) infested tree equilibria for increasing values of n; f) proportion of local firewood strategist equilibria for increasing values of n.

When the strength of social norms *n* is increased above baseline, the proportion of local strategists drops to zero more quickly for all tested values of *n* (Fig. 2) up to *n* = 10 (not shown). Increasing the strength of social norms causes increased conformity to the strategy of the majority–the transport strategists under conditions of *L_1_*(0) = *L_2_*(0) = 0.1–thus there is no incentive to become a local strategist. At these parameter values, the effect of increasing the strength of social norms is to reinforce the pre-existing tendency of the population, hence, no changes can be seen in the equilibrium number of susceptible or infested trees compared to the baseline case (Fig. 2d–f).

### Increasing the Imitation Rate

**Figure 3:**
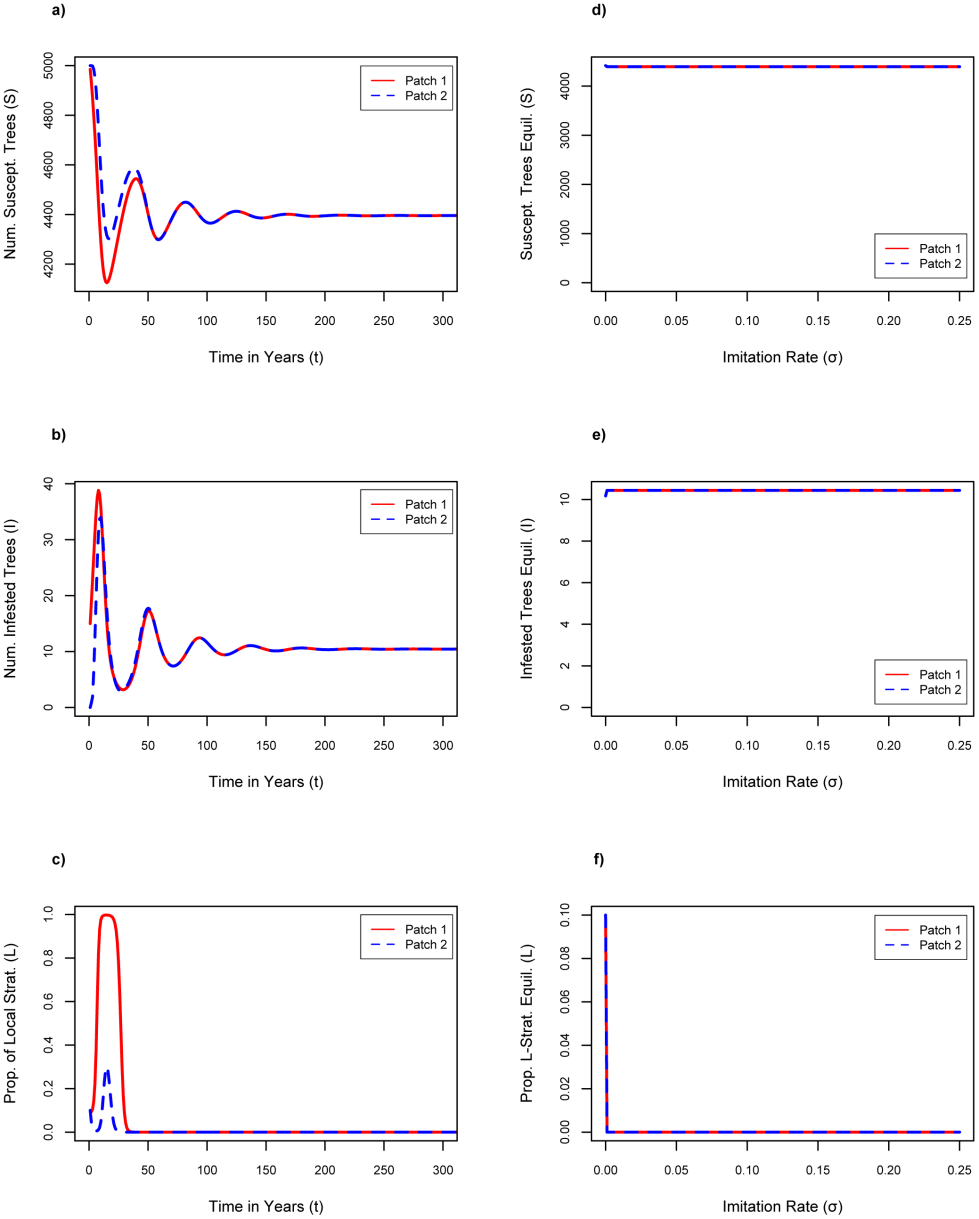
Results of increasing imitation rate. Increase imitation rate: n = 0.1, f = 0.1, Cl = $6.75 where a) number of susceptible trees as a function of time with σ = 0.5; b) number of infested trees as a function of time with σ = 0.5; c) proportion of local firewood strategists as a function of time with σ = 0.5; d) susceptible tree equilibria for increasing values of σ; e) infested tree equilibria for increasing values of σ; f) proportion of local firewood strategist equilibria for increasing values of σ.

Increasing the imitation rate *σ* allows the *L*_1_–the proportion of local strategists in patch 1–to jump to 100%, at least momentarily (compare Fig. 3c to Fig. 1c). A higher imitation rate enables a faster conversion of transport strategists to local strategists during the worst period of the outbreak in patch 1, from *t = *1 to 3 years. However, as the infestation in patch 1 falls back to its lower, endemic level, the infestation-induced pressure to adopt the local strategy disappears, and *L*_1_ eventually falls back to zero due to the continuing lower cost of transporting firewood. In contrast, *L*_2_ peaks at 0.3 before falling back to zero, because the infestation in patch 2 is less severe in the first few years. The equilibrium proportion of local strategists is zero for all values of *σ* (Fig. 3) up to *σ = *0.99 (not shown), since social learning, like social pressure, tends to reinforce the pre-existing tendencies of the population. Likewise, the equilibrium number of susceptible or infested trees does not change significantly compared to the baseline case (Fig. 3d, e). Higher imitation rates cause individuals to be more likely to convert to the strategy of the majority, which in our experiment is the transport strategists, thus there is no pressure to become a local strategist, except during the periods when infestation is severe.

### Increasing the Infestation Concern

**Figure 4:**
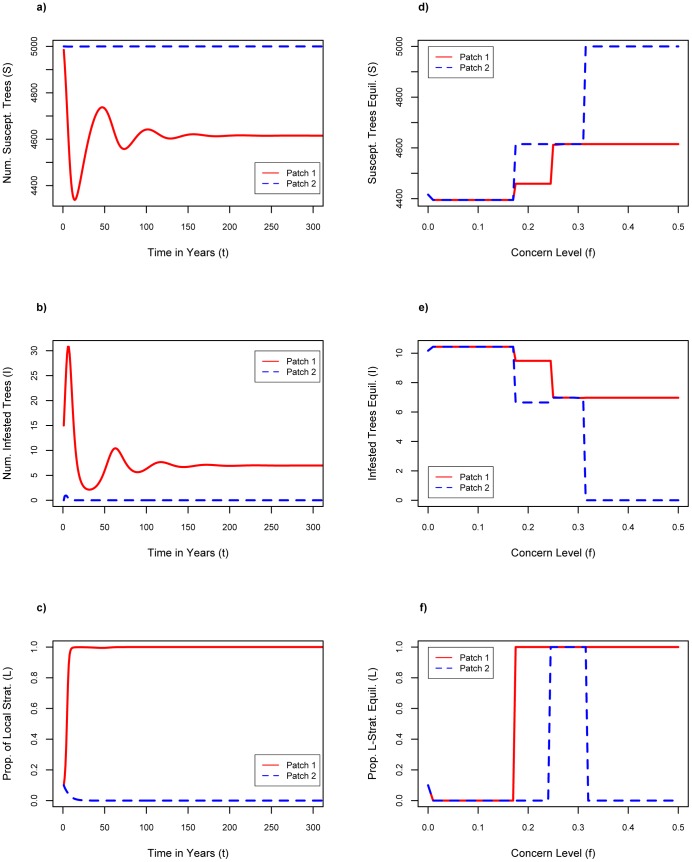
Results of increasing concern of infestation. Increase concern of infestation: n = 0.1, σ = 0.1, Cl = $6.75 where a) number of susceptible trees as a function of time with f = 0.35; b) number of infested trees as a function of time with f = 0.35; c) proportion of local firewood strategists as a function of time with f = 0.35; d) susceptible tree equilibria for increasing values of f; e) infested tree equilibria for increasing values of f; f) proportion of local firewood strategist equilibria for increasing values of f.

Increasing *f*, the level of concern caused by infestations, has a significant effect on dynamics (Fig. 4). A sufficiently large value of *f* can prevent the spread of the infestation from patch 1 to patch 2 (Fig. 4a,b) as well as the permanent transition of individuals in patch 1 to local strategists, who thereby prevent spread of the pest from patch 1 to patch 2 (Fig. 4c). This change in outcomes can also be seen in figures showing the equilibrium number of susceptible trees, infested trees and local strategists in both patches, versus *f* (Fig. 4d–f). There are two sharp thresholds in these plots: when *f* ≤0.17, strong cross-patch infestation occurs, the pest becomes endemic, and all individuals are local strategists, as in the baseline scenario; when 0.17< *f* <0.3113, moderate crosspatch infestation occurs and *L_1_* = 1 while *L_2_* remains low until *f* = 0.25 after which *L_2_* = 1 as well (Fig. 4f); finally, when *f* >0.3113 crosspatch infestation is eliminated and *I_1_* equilibrates to approximately 7 while *I_2_* equilibrates to 0 and *L_1_* goes to 1 while *L_2_* drops to 0 (See Fig. 4e and 4f), corresponding to a scenario where individuals in patch 1 prevent spread of the infestation to patch 2. Interestingly, when 0.25< *f* <0.3113, *L_1_* = *L_2_* = 1; at these parameter values, there is enough infestation in patch 2 for that population to become dominated by local strategists as well.

### Decreasing the Cost of Local Firewood

**Figure 5:**
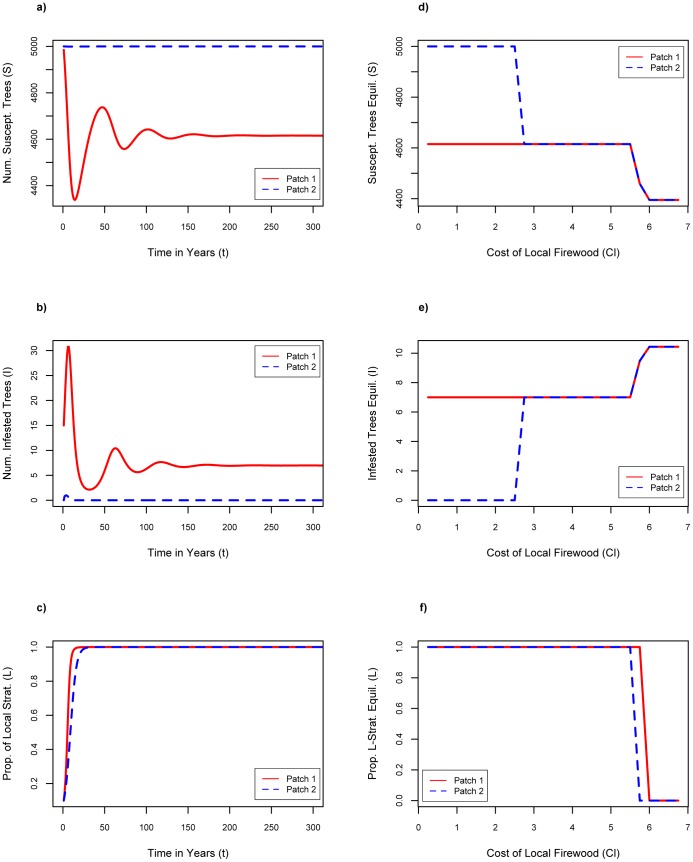
Results of decreasing cost of local firewood. Decrease cost of local firewood: n = 0.1, f = 1, σ = 0.1 where a) number of susceptible trees as a function of time with Cl = $2.25; b) number of infested trees as a function of time with Cl = $2.25; c) proportion of local firewood strategists as a function of time with Cl = $2.25; d) susceptible tree equilibria for increasing local firewood costs; e) infested tree equilibria for increasing local firewood costs; f) proportion of local firewood strategist equilibria for increasing local firewood costs.

Decreasing the cost of local firewood, *C_l_*, also has a significant impact on dynamics (Fig. 5). When the cost drops sufficiently from baseline, spread of infestation to patch 2 is prevented and individuals in both patches become local strategists (Fig. 5a–c). Thresholds again appear as *C_l_* is varied. When *C_l_* is decreased below $5.75, adherence to the local strategy at equilibrium increases to 100% in both patches regardless of the infestation level (even though transported firewood is still cheaper, *C_t_* = $5.00). When *C_l_* ≤ $2.25, crosspatch infestation is completely eliminated, even for low values of the other parameters of social influence (Fig. 5e), because buying firewood locally is now sufficiently economically beneficial to individuals, and the difference in utility is large enough such that local strategists in patch 1 dominate quickly enough to prevent spread of the infestation to patch 2.

### Increasing Infestation Concern while Decreasing Cost of Local Firewood

**Figure 6:**
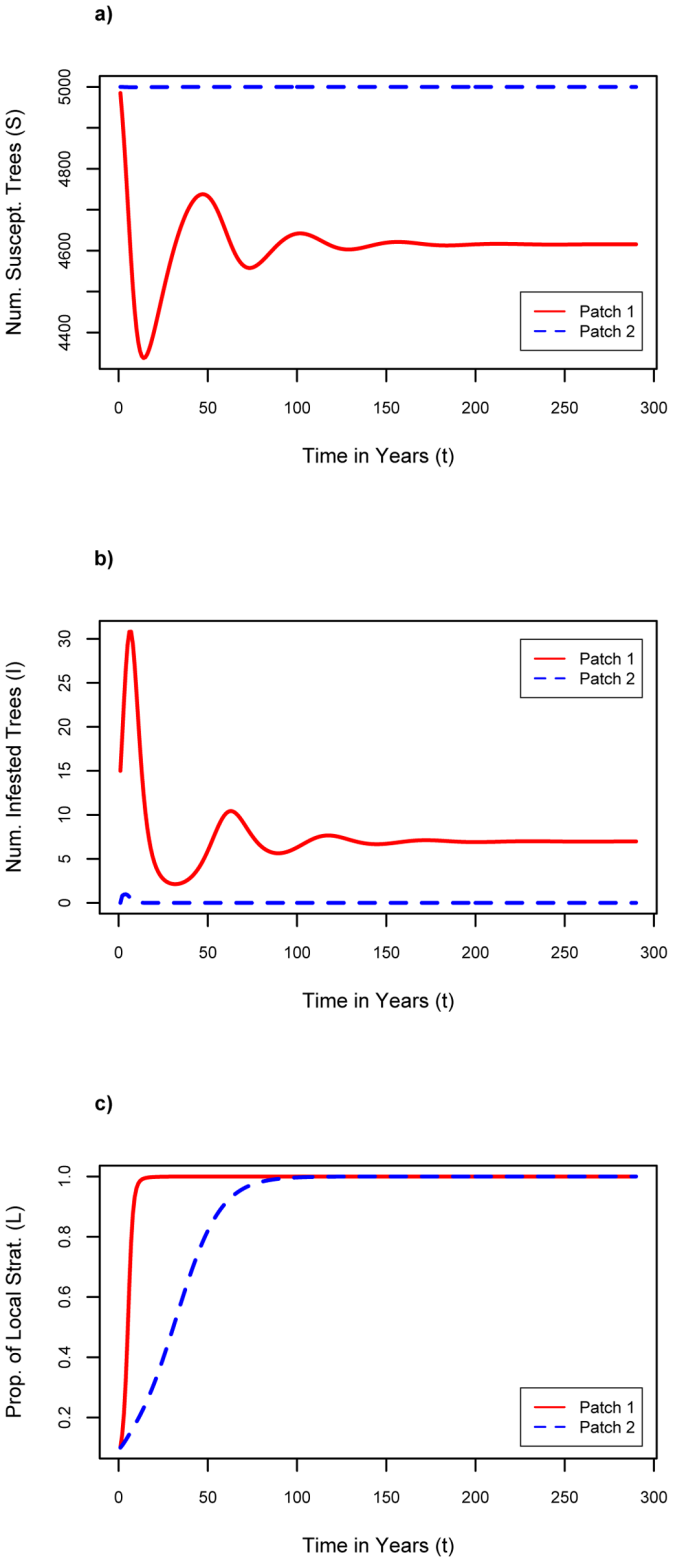
Results of increasing concern while decreasing cost of local firewood. Increase concern while decreasing cost: n = 0.1, σ = 0.1, where a) number of susceptible trees as a function of time with f = 0.2, Cl = $4.25; b) number of infested trees as a function of time with f = 0.2, Cl = $4.25; c) proportion of local firewood strategists as a function of time with f = 0.2, Cl = $4.25.

**Figure 7:**
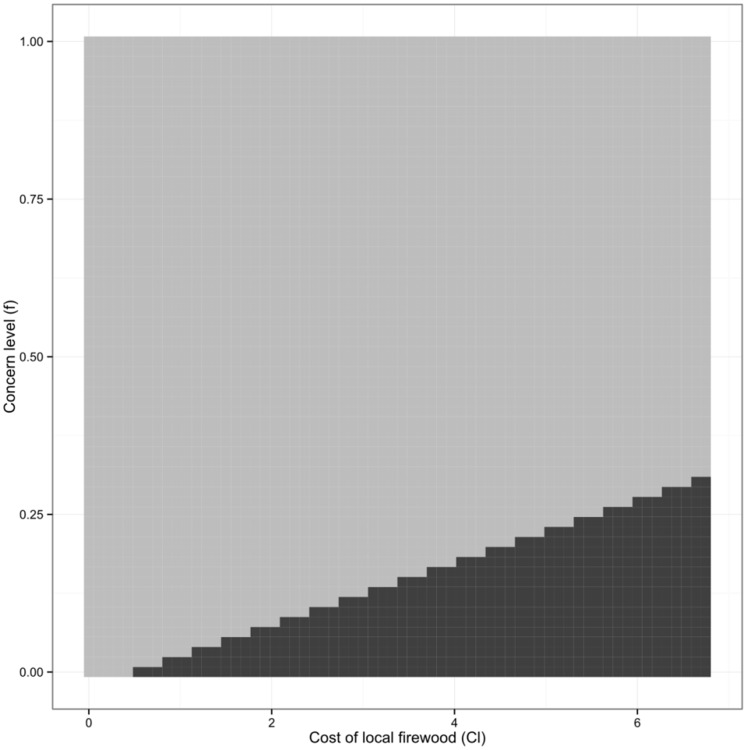
Phase portrait of relationship between concern and cost of local firewood. Phase portrait for simultaneous changes to concern level (f) and local cost of firewood (Cl). The dark region represents containment of the infestation while the light region represents continued spread.

When the level of concern *f* is simultaneously increased while decreasing the local cost of firewood *C_l_*, much smaller changes are needed to each parameter in order to prevent the spread of the pest to the healthy patch. An example time series appears in Fig. 6; in this case, the level of concern is increased to a value that is insufficient on its own to prevent cross-patch infestation (*f* = 0.2) and the cost of local firewood is simultaneously decreased to a price that is also insufficient alone to prevent cross-patch infestation (*C_l_ = *$4.25), however we see that the combined effects are enough to eliminate crosspatch infestation. Similar results are found when *f* = 0.175 and *C_l_ = *$4.00, but not for all lower combinations. The *f-C*_l_ parameter space is explored in Fig. 7, showing the boundary between the elimination and no elimination outcomes for a range of values of *f* and *C*_l_. Hence, a combination of increasing the attractiveness of the local strategy through both public awareness (increasing *f*) and making local purchase of firewood more economically attractive (decreasing *C_l_*) may be more effective than either in isolation.

## Discussion

Here we used a model of a coupled human-environment forest pest system to show the complex dynamics that can emerge from such systems, and to explore strategies to prevent spread of infestation between patches, when control efforts depend on collective individual choices not to transport firewood. The rich possibilities for dynamics come from the interplay between the natural system dynamics of forest pest invasions, social pressure to conform, social learning rates, economic costs of the two strategies, and concern regarding causing an infestation. For example, social pressure can force the population to become dominated by either local or transport strategists, depending on which are initially numerous. However, if concern for infestation is high enough, an infestation could force the population temporarily into the regime of predominant local strategists, and if social pressure is strong enough and social learning is operating quickly enough, social pressure could then make that temporary transition permanent. Economic incentives can change the threshold at which this transition occurs.

With a solely education-based strategy (increasing the value of *f*), crosspatch infestation can be eliminated for high enough values of *f*, but the proportion of local strategists in the healthy patch drops to zero when infestation is no longer a concern (see Fig. 4c). This allows for firewood transport to begin again, subsequently increasing the risk of introducing a new population of insects. Therefore this method could be effective only if there is a very small ideal level of concern where 0.25< *f* <0.3113. It would likely be difficult to ensure that levels of concern remained in such a small interval. Alternatively, reducing the cost of local firewood is a very effective management strategy; however, this may represent a fairly high economic cost to the government. When costs are reduced with no other changes to parameter values, the price of locally purchased firewood must drop from $6.75 to at least $2.25 (Fig. 5), which amounts to a $4.25 subsidization per bundle of firewood. However, by pairing reduced costs with increased concern, we see that the level of concern does not need to be as high to eliminate crosspatch infestation and less subsidization would be required. Increasing *f* to 0.175 and decreasing *C_l_* to $4.00, or increasing *f* to 0.2 and decreasing *C_l_* to $4.25, are very effective strategies that prevent cross-patch infestation and increase adherence to the local strategy to 100% over time (Fig. 6). We can see from Fig. 7 that there is a linear relationship between the threshold values of *f* and *C_l_* for elimination. Hence, one approach to optimizing pest control strategy would be to choose an acceptable level of subsidization and then work to increase the level of concern to the necessary degree in order to contain the spread.

Most previous models have focused on infestation in a patch where the pests have already been introduced, and have not taken human behavioural feedback into account [8]–[14]. In contrast, here we considered the problem of preventing spread between patches through public adherence to control measures. For this problem, cooperation from the public through voluntary adherence to firewood movement restrictions is crucial. Including behavioural feedback allowed us to examine the two-way interplay between human decisions and actions and the environmental state. The resulting model showed that [1] concern about spreading infestation and [2] the economic cost of local firewood significantly determine the feasibility of controlling spread. It is possible to adjust these parameters in real populations through education campaigns (*f*) and the subsidization of local firewood in provincial and national parks (*C*_l_). Moreover, our results indicate that a highly effective approach for preventing both ALB and EAB spread to other patches might involve a combination of these two strategies. This would provide the time needed to develop an elimination strategy in a given patch, such as using insecticide.

The finding that making locally purchased firewood (*C*_l_) cheaper than transported firewood (*C*_t_) can be an effective means of control perhaps comes as no surprise. However, the finding that increasing the concern of infestation (*f*) can be effective is perhaps more surprising, due to the fact that better control leads to a decrease in the infestation level *I*_i_, and hence a decrease in the pressure due to infestation concern (*fI*_i_). In short, it is possible for complacency to threaten the effectiveness of a strategy that relies upon current infestation as a motive. However, in this case, the effectiveness of infestation concern was aided by the fact that a high level of concern in patch 1 prevented spread to patch 2 (not patch 1), and also that, once infestation enabled the population to temporarily reach a point where local strategists dominated, social norms could then take over to ensure fixation of the population in that state.

The most important limitations of the current model include its deterministic nature and the simplicity of the assumed social structures. Although we included a term *Φ(I_i_)* to partially capture effects relating to stochasticity and strong Allee effects, other sources of stochasticity (e.g. climate variation, demographic stochasticity) were not included. Furthermore, the psychology of decision-making and motivation is a complex field that encompasses a variety of individual and social factors [19]–[21]. Simple representations of social processes are often sufficient for certain model applications [20], hence our motivation for keeping the model of social processes relatively simple. However, including factors such as social heterogeneity could change dynamics. Additionally, our current model is only a two-patch system, but an extension to a multi-patch system would better capture the complexities of regional forest pest control in a large system of provincial and federal parks.

For the sake of simplicity in the model, the carrying capacity (*K_i_*) was assumed to be the same in each patch. While it is likely that differences in the carrying capacity would affect the results in terms of whether the infestation were sustained and how long it would take to reach equilibrium, we believe that our conclusions would still hold. Each patch is of arbitrary size and in part defined by the carrying capacity itself. Thus, a change in the carrying capacity in patch i would reflect a change in the size of patch *i*. The specifics of patch size would require significantly more parameterization to the environmental conditions of a specific region and therefore left for future research.

The current policy in many North American jurisdictions is self-regulated quarantine in areas with known ALB or EAB infestation; signs are posted requesting that people do not bring unfinished wood products out of the area. While businesses and government organizations are checked for adherence to these regulations, individuals are not (personal communication, CFIA). As such, most individuals are unaware of the risk of transporting firewood (D.M. Runberg, unpublished report, 2011). According to our limited survey of firewood costs, firewood is generally cheaper to purchase outside of government parks, and so many people will purchase the firewood they require wherever is most convenient for them and then transport it to their final destination. Additionally, there is no regulation of the source of firewood for private sale, so firewood purchased privately near to the final destination may, in fact, still not be local. In contrast, firewood sold inside parks is always local. Hence, there is scope to apply a strategy of subsidizing the cost of firewood sold by parks. The tendency for individuals to purchase firewood privately could be addressed in future firewood pricing policy in the parks, as it has been shown here that the economic cost of local firewood makes a significant impact on the rate of spread for the ALB and EAB. Future budget impact or cost-effectiveness analysis would enable determining the optimal combined strategy of firewood subsidization (*C*_l_) and public education (*f*) to minimize the risk of cross-patch dispersal.

## Conclusions

In a coupled human-environment system model for mitigating cross-patch dispersal of Asian longhorned beetle and emerald ash borer through voluntary firewood movement restrictions, effective management strategies involve increasing the level of concern regarding infestations, and/or decreasing the cost differential between locally purchased and transported firewood. Furthermore, synergies between strategies are possible: moderate subsidization of locally available firewood paired with low to moderate increase in infestation concern through education can be a highly effective strategy.
